# Alleviating the Adverse Effects of Lipopolysaccharide on Production Performance, Immune Function, and Intestinal Damage in Broilers via Dietary Alkaline Phosphatase

**DOI:** 10.3390/ani16101525

**Published:** 2026-05-16

**Authors:** Qijun Wang, Ying Zhou, Quan Qiu, Wei Zhang, Shahid Ali Rajput, Desheng Qi, Minggang Lei

**Affiliations:** 1 College of Animal Science and Technology, Huazhong Agricultural University, Wuhan 430070, China; wangqijun@webmail.hzau.edu.cn; 2 Wuhan Sunhy Biology Co., Ltd., Wuhan 430070, China; tech@sunhy.cn (Y.Z.); quanqiu@webmail.hzau.edu.cn (Q.Q.); zhangw8804@163.com (W.Z.); 3 Department of Animal and Dairy Sciences, Faculty of Veterinary and Animal Sciences, Muhammad Nawaz Shareef University of Agriculture, Multan 66000, Pakistan; shahid.ali@mnsuam.edu.pk

**Keywords:** alkaline phosphatase, broilers, lipopolysaccharide, intestinal damage, tight junction proteins

## Abstract

In this study, we investigated how adding alkaline phosphatase (ALP) to the diet improves the growth performance and intestinal function of broiler chickens under lipopolysaccharide (LPS) stress. A total of 280 one-day-old broilers were randomly allocated into four groups, each consisting of five replicates with 14 birds per replicate, and reared for 42 days. The control group and the LPS group were fed a basal diet, whereas the LPS + 1000 U/kg ALP group and the LPS + 5000 U/kg ALP group were fed the basal diet supplemented with 1000 and 5000 U/kg of ALP, respectively. Except for the control group, the other three groups were intraperitoneally injected with LPS solution at a dose of 500 μg/kg body weight at ages of 15, 17, 19, 23, 25, and 27 days. After the data were analyzed by one-way analysis of variance (ANOVA), the results showed that LPS significantly inhibited growth, disrupted intestinal structure, upregulated inflammatory genes, and downregulated tight junction protein expression, whereas dietary supplementation with 1000 U/kg ALP significantly alleviated these adverse effects, restored intestinal structure, enhanced antioxidant capacity, and modulated related gene expression. In summary, dietary ALP supplementation alleviated LPS-induced growth inhibition and intestinal damage in broilers.

## Simple Summary

In this study, we investigated how dietary supplementation with alkaline phosphatase (ALP) alleviates lipopolysaccharide (LPS)-induced stress responses in broilers. LPS stress reduces growth performance, damages intestinal structure, increases inflammation, and impairs intestinal barrier function in broilers. In the present study, we found that dietary supplementation with ALP, particularly at the dose of 1000 U/kg, effectively reversed these negative effects. ALP improved growth performance, restored intestinal morphology, reduced intestinal inflammation, and strengthened intestinal barrier function in broilers. These results indicate that ALP can serve as a functional feed additive to protect broilers against LPS-induced stress.

## 1. Introduction

In the context of intensive farming, broiler chickens face multiple challenges such as high-density feeding, environmental stress, pathogenic bacterial infections, and poor feed hygiene [1,2]. These factors can induce excessive activation of the immune system (immune stress), leading to growth inhibition, impaired intestinal barrier function, and increased susceptibility to diseases, causing significant economic losses to the broiler farming industry [2,3]. Therefore, exploring nutritional regulation strategies to alleviate immune stress has important scientific significance and production application value.

Lipopolysaccharide (LPS) is an important structural component of the outer membrane of Gram-negative bacteria and a key pathogen-related molecular pattern that induces immune responses in the body [4]. It can activate downstream inflammatory signaling pathways such as NF-κB by binding to toll-like receptor 4 (TLR4), inducing the release of a large number of pro-inflammatory cytokines, thereby triggering systemic inflammatory responses, oxidative stress, and intestinal barrier dysfunction [4,5]. Numerous studies have shown that intraperitoneal injection of LPS can simulate systemic inflammatory responses induced by bacterial infection, leading to decreased feed intake, decreased nutrient utilization, growth inhibition, and damage to intestinal structure in broiler chickens [3,6,7]. Due to its clear mechanism of action and stable and controllable model, LPS is widely used as a model inducer for acute immune stress in poultry research [3,6,7].

The intestine is not only the main location for nutrient digestion and absorption, but also the first line of defense for the body to resist invading exogenous pathogens [8]. Under LPS stimulation, the intestinal morphology and structure are damaged, manifested as villus atrophy, crypt proliferation, and decreased villus-to-crypt ratio [3,9]. These structural changes are closely related to increased intestinal permeability and translocation of pathogens and toxins within the intestinal lumen, thereby exacerbating local and systemic inflammatory responses [10,11]. Meanwhile, LPS can also disrupt the expression of tight junction proteins in the intestine, damaging the integrity of the intestinal barrier [12,13]. In addition, LPS stress upregulates the expression of pro-inflammatory cytokines such as IFN-γ and IL-17, which not only directly mediate inflammatory responses but also exacerbate intestinal damage by affecting epithelial cell proliferation and apoptosis [6,14,15].

Alkaline phosphatase (ALP) is a membrane-bound glycoprotein widely present in the intestinal, liver, and skeletal tissues of mammals and poultry [16]. Among them, intestinal alkaline phosphatase (IAP) is located on the brush border membrane of intestinal epithelial cells, and one of its core functions is to neutralize LPS through dephosphorylation [17,18]. IAP can hydrolyze the phosphate groups in the lipid A region of LPS, block their binding to TLR4, and thereby inhibit the cascade amplification of downstream inflammatory signals [19]. In addition, ALP also participates in maintaining intestinal microbiota homeostasis, regulating the pH of the intestinal environment, and promoting intestinal barrier repair, playing multiple protective roles in maintaining intestinal homeostasis [20,21,22]. In recent years, dietary ALP supplementation has been shown to effectively improve intestinal inflammation and enhance intestinal barrier function in animal models of colitis and bacterial infections [23,24]. However, in the field of poultry, especially in response to LPS-induced intestinal injury and growth inhibition, the regulatory role of dietary ALP is still lacking systematic research, and its potential application in broiler production has not been fully explored.

Therefore, in this study, we aim to establish an immune stress model in broiler chickens by intraperitoneal injection of LPS, and systematically evaluate the effects of adding different doses of ALP to the diet on the production performance, intestinal morphology, antioxidant capacity, inflammatory factor expression, and tight junction protein expression of broiler chickens, in order to provide a theoretical basis for the application of ALP as a new type of anti-stress feed additive in broiler production.

## 2. Materials and Methods

### 2.1. Experimental Design and Dietary Treatment

The experimental protocol in this study has been approved by the Animal Ethics Committee (Ethics Review Number: HZAUCH-2026-0027). In this experiment, we selected 280 healthy white feathered broiler chickens (Arbor Acres (AA+)) at one day of age and randomly divided them into four treatment groups based on the principle of similar initial body weight—control, LPS, low-dose ALP (LPS + 1000 U/kg ALP), and high-dose ALP (LPS + 5000 U/kg ALP)—with a total of five replicates in each group and 14 broiler chickens (half male and half female) in each replicate. The control and LPS groups were fed with a basal diet, while the low- and high-dose ALP groups of broiler chickens had 1000 and 5000 U/kg of ALP added to their basal diets, respectively. The basal diet of broiler chickens is formulated according to the recommended amount in NRC proposed in 1994, divided into two stages: 1–21 and 22–42 days. The composition and nutritional level of the raw materials are shown in Table 1. At 8:00 am on the 15th, 17th, 19th, 23rd, 25th, and 27th days of the experiment, broiler chickens were weighed on an empty stomach. LPS solution at 500 μg/kg body weight was injected into the abdominal cavity of broiler chickens in the LPS group and low- and high-dose ALP groups, while the control group was injected with an equal amount of sterile physiological saline. ALP was provided by Sunhy Biology Co., Ltd. (Wuhan, China) with an enzyme activity of 5000 U/g, and LPS (from *Escherichia coli* serotype O55: B5) was purchased from Sigma-Aldrich (St. Louis, MO, USA), catalog number L2880.

### 2.2. Feeding Management and Production Performance Statistics

This experiment was conducted in the broiler house of Sunhy Tuanfeng Experimental Base, with a testing period of 42 days. All broiler chickens are raised in stainless steel cages (dimensions: 100 cm × 80 cm × 40 cm, length × width × height) within semi-open chicken houses, using a single-floor flat rearing method. They were exposed to 24-hour light provided by a combination of artificial and natural lighting. During the entire experimental period, broiler chickens were allowed to freely feed and drink water. Broilers were kept under a controlled temperature schedule: 33 °C for the first three days, then reduced by 2–3 °C each week until reaching 22 °C at the end of the trial. The relative humidity was maintained between 55% and 65%. During the experiment, the daily feed intake was recorded, and the broiler chickens were weighed on days 0, 21, and 42. Based on the obtained data, the average daily weight gain, average daily feed intake, and feed conversion ratio of each group of broiler chickens were calculated for days 1–21 and 22–42.

### 2.3. Sample Collection

After the feeding periods on days 21 and 42, one broiler with a body weight close to the average value was randomly selected from each replicate, and blood was collected from the brachial (wing) vein. The blood samples were centrifuged at 3000 r/min for 15 minutes to isolate the serum, which was then aliquoted and stored in a freezer at −80 °C for subsequent determination of serum immunoglobulin levels. After blood collection, the broilers were euthanized by cervical dislocation, then immediately dissected, and each intestinal segment was separated. The jejunal mucosa was scraped into cryotubes, rapidly frozen in liquid nitrogen, and then stored at −80 °C for subsequent detection of antioxidant indices and gene expression. Approximately 1 cm sections of the duodenum, jejunum, and the middle part of the ileum were collected and temporarily preserved in 4% paraformaldehyde solution for the preparation of paraffin sections to examine morphological changes in each intestinal segment.

### 2.4. Determination of Serum Immunoglobulin Levels

The levels of IgA, IgG, and IgM in the serum of broiler chickens were measured using enzyme-linked immunosorbent assay (ELISA) kits (Nanjing Jiancheng Bioengineering Institute, Nanjing, China). All procedures were performed strictly in accordance with the manufacturer’s instructions.

### 2.5. Determination of Antioxidant Indices in Jejunal Mucosa

The activities of T-AOC, SOD, GSH-Px, and CAT and the content of MDA in the jejunal mucosa were determined using commercial kits (Nanjing Jiancheng Bioengineering Institute, Nanjing, China). All procedures were performed strictly in accordance with the manufacturer’s instructions.

### 2.6. Observation of Intestinal Morphology

After fixation with 4% paraformaldehyde solution, the duodenum, jejunum, and ileum tissues were sequentially dehydrated with ethanol, transparent with xylene, and embedded in paraffin to prepare 5 μm thick paraffin tissue sections. The sections were then stained with hematoxylin and eosin (H&E). The morphological structure of each intestinal tissue was observed using a light microscope (Olympus Corporation, Tokyo, Japan). The villus height and crypt depth of each intestinal segment were measured using Image-Pro Plus 6.0 image analysis software (Media Cybernetics, Inc., Rockville, MD, USA), and the villus-height-to-crypt-depth ratio (V/C ratio) was calculated. Five non-overlapping fields of view were randomly selected from each sample for quantitative analysis.

### 2.7. Extraction of mRNA from Jejunal Mucosa and RT-qPCR

The jejunal mucosal tissue was removed from the −80 °C freezer, and total RNA was extracted from it using TRIzol reagent (Thermo Fisher Scientific, Waltham, MA, USA). Reverse transcription reagent (ABclonal Technology Co., Ltd., Wuhan, China) was used to remove genomic DNA and perform reverse transcription. RT-qPCR was performed using fluorescent quantitative reagent (ABclonal Technology Co., Ltd., Wuhan, China) on a real-time fluorescent quantitative PCR instrument (Bio-Rad Laboratories, Inc., Hercules, CA, USA). Each step of the operation was strictly carried out according to the instructions of the reagent. The primers for RT-qPCR are shown in Table 2. Using *β-actin* as an internal reference gene, relative quantitative analysis of the expression level of the target gene was performed using the 2^−ΔΔCt^ method.

### 2.8. Data Processing and Statistical Analysis

Experimental data were initially collated and summarized using Microsoft Excel 2016, and SPSS 26.0 statistical software (IBM Corp., Armonk, NY, USA) was used for data analysis. The data of each group is expressed as mean ± standard deviation (Mean ± SD) (*n* = 5). One-way ANOVA was used to test the overall differences between groups. When the differences between groups were significant, Duncan’s method was used for multiple comparisons. The criterion for determining statistical significance of differences is *p* < 0.05.

## 3. Results

### 3.1. Ameliorative Effects of Dietary Alkaline Phosphatase on Growth Performance of Broilers Under Lipopolysaccharide Stress

The effects of different treatments on the production performance of broiler chickens at different stages are shown in Table 3. The initial weight of broiler chickens was around 40 g, and there was no significant difference (*p* > 0.05), indicating uniform grouping. At the age of 1–21 days, the final weight, average daily feed intake, and average daily gain of the LPS group were significantly reduced (*p* < 0.05) compared with the control group. Compared with the LPS group, the low-dose ALP group showed a significant increase in final weight and average daily gain (*p* < 0.05), while the high-dose ALP group showed an increasing trend in final weight and average daily gain (*p* > 0.05). At the age of 22–42 days, the final weight, average daily feed intake, and average daily gain of the LPS group were significantly lower than those of the control group (*p* < 0.05); compared with the LPS group, those of the low- and high-dose ALP groups were significantly increased (*p* < 0.05), and there was no significant difference compared with the control group (*p* > 0.05). From the entire experimental phase (1–42 days), the average daily feed intake and average daily gain of the LPS group were significantly lower than those of the control group, with reductions of 9.71% and 11.59%, respectively (*p* < 0.05); compared with the LPS group, those of the low- and high-dose ALP groups were significantly increased (*p* < 0.05), and there was no significant difference compared with the control group (*p* > 0.05). Throughout the entire experimental period, different treatments had no significant effect on the feed conversion ratio of broiler chickens (*p* > 0.05).

### 3.2. Effects of Dietary Alkaline Phosphatase on Serum Immunoglobulin Levels in Broilers Under Lipopolysaccharide Stress

The effects of different treatments on serum immunoglobulin levels in broiler chickens are shown in Table 4. On the 21st day, compared with the control group, the levels of immunoglobulin A (IgA) and immunoglobulin G (IgG) in the serum of the LPS group decreased by 17.19% and 37.39%, respectively, while that of immunoglobulin M (IgM) increased by 60.24%. However, the differences between the groups did not reach a statistically significant level (*p* > 0.05). Compared with the LPS group, the high-dose ALP group showed an increasing trend in IgA levels and a decreasing trend in IgM levels in serum, but the difference in changes was also not significant (*p* > 0.05). On the 42nd day, there was no significant difference in the levels of IgA, IgM, and IgG in the serum of each group (*p* > 0.05).

### 3.3. Effects of Dietary Alkaline Phosphatase on Antioxidant Enzyme Activities in the Jejunal Mucosa of Broilers Under Lipopolysaccharide Stress

The effects of different treatments on antioxidant enzyme activity in the jejunal mucosa of broiler chickens are shown in Table 5. On the 21st day, compared with the control group, the glutathione peroxidase (GSH-Px) activity in the jejunal mucosa of the LPS group decreased by 24.94%, but the difference in changes was not significant (*p* > 0.05). The malondialdehyde (MDA) content significantly increased by 136.96% (*p* < 0.05), indicating that LPS may induce an oxidative stress response. Compared with the LPS group, the GSH-Px activity in the jejunal mucosa of the high-dose ALP group showed an increasing trend, while the MDA content in the low-dose ALP group showed a decreasing trend, but the differences in changes were not significant (*p* > 0.05). On the 42nd day, there were no significant changes in the activities of antioxidant enzymes and MDA content in the LPS group (*p* > 0.05) compared with the control group; compared with the LPS group, the activity of catalase (CAT) was significantly increased in the low-dose ALP group, and the activity of GSH-Px was significantly increased in the high-dose ALP group (*p* < 0.05).

### 3.4. Effects of Dietary Alkaline Phosphatase on the Duodenal Villus Structure of Broilers Under Lipopolysaccharide Stress

**Figure 1:**
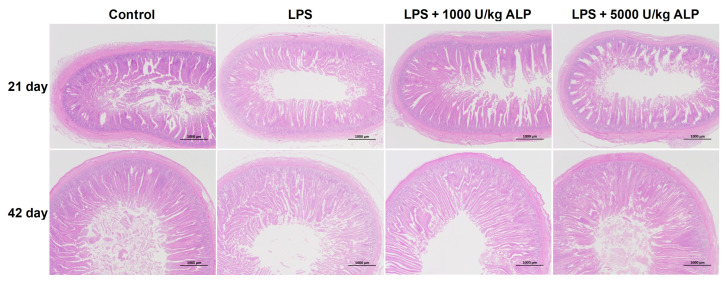
Histological section of the duodenum.

As shown in Figure 1, LPS induced duodenal damage, while ALP supplementation partially restored the structure. The effects of different treatments on the villus structure of the duodenum in broiler chickens are shown in Table 6. On the 21st day, compared with the control group, the crypt depth of the duodenum in the LPS group significantly increased by 28.23% (*p* < 0.05), the villus-to-crypt ratio significantly decreased by 36.64% (*p* < 0.05), and the villus height showed a decreasing trend (18.31%) (*p* > 0.05). Compared with the LPS group, the villus height of the duodenum in both low- and high-dose ALP groups showed an increasing trend (*p* > 0.05), and the crypt depth was significantly reduced (*p* < 0.05). The villus-to-crypt ratio in the low-dose ALP group was significantly increased (*p* < 0.05), while that in the high-dose ALP group showed an increasing trend, but the difference in changes was not significant (*p* > 0.05). On the 42nd day, compared with the control group, the LPS group showed a slight decrease in the villus height and villus-to-crypt ratio of the duodenum (5.01% and 15.44%, respectively), and a slight increase in crypt depth (11.19%), but the differences between the groups did not reach a statistically significant level (*p* > 0.05).

### 3.5. Effects of Dietary Alkaline Phosphatase on the Jejunal Villus Structure of Broilers Under Lipopolysaccharide Stress

**Figure 2:**
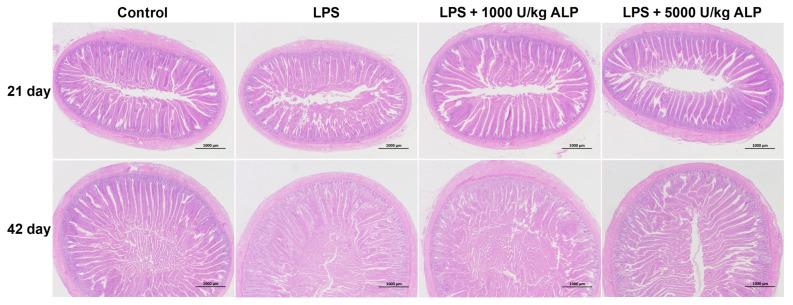
Histological section of the jejunum.

As shown in Figure 2, LPS induced jejunal damage, while ALP supplementation partially restored the structure. The effects of different treatments on the villus structure of broiler jejunum are shown in Table 7. On the 21st day, compared with the control group, the crypt depth of the jejunum in the LPS group significantly increased by 38.87% (*p* < 0.05), the villus-to-crypt ratio significantly decreased by 35.27% (*p* < 0.05), and the villus height showed a downward trend (decreased by 9.84%, but not statistically significant) (*p* > 0.05). Compared with the LPS group, the crypt depth of the jejunum in the low- and high-dose ALP groups was significantly reduced by 25.87% and 22.57%, respectively (*p* < 0.05), and the villus-to-crypt ratio was significantly increased by 50.20% and 44.29%, respectively (*p* < 0.05). On the 42nd day, compared with the control group, the crypt depth of the jejunum in the LPS group significantly increased by 24.48% (*p* < 0.05), while the villus height and villus-to-crypt ratio showed a decreasing trend (7.32% and 25.90%, respectively), but the difference in changes was not significant (*p* > 0.05). Compared with the LPS group, the crypt depth of the jejunum in the low- and high-dose ALP groups decreased, while the villus height and villus-to-crypt ratio increased, but the differences in changes were not significant (*p* > 0.05).

### 3.6. Effects of Dietary Alkaline Phosphatase on the Ileal Villus Structure of Broilers Under Lipopolysaccharide Stress

**Figure 3:**
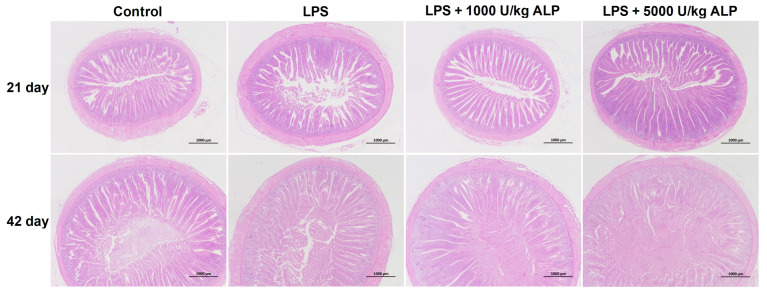
Histological section of the ileum.

As shown in Figure 3, LPS induced ileal damage, while ALP supplementation partially restored the structure. The effects of different treatments on the villus structure of the ileum in broiler chickens are shown in Table 8. On the 21st day, compared with the control group, the villus height of the ileum in the LPS group was significantly reduced by 26.44%, the crypt depth was significantly increased by 33.15%, and the villus-to-crypt ratio was significantly reduced by 44.83% (*p* < 0.05). Compared with the LPS group, the villus height of the ileum in the low- and high-dose ALP groups significantly increased by 58.13% and 60.87%, respectively; the crypt depth significantly decreased by 30.11% and 18.18%, respectively; and the villus-to-crypt ratio significantly increased by 126.28% and 96.68%, respectively (*p* < 0.05). The villus height, crypt depth, and villus-to-crypt ratio of the ileum in the low- and high-dose ALP groups were restored to levels close to those of the control group. On the 42nd day, compared with the control group, the villus-to-crypt ratio of the ileum in the LPS group was significantly reduced by 24.61% (*p* < 0.05), and there was no significant difference in the changes in villus height and crypt depth (*p* > 0.05). Compared with the LPS group, the villus-to-crypt ratio of the ileum was significantly increased in the high-dose ALP group (*p* < 0.05), while there was no significant difference in the changes in the low-dose ALP group (*p* > 0.05).

### 3.7. Effects of Dietary Alkaline Phosphatase on the Gene Expression Levels of Inflammatory Cytokines in the Jejunal Mucosa of Broilers Under Lipopolysaccharide Stress

The effects of different treatments on the gene expression levels of inflammatory factors in the jejunal mucosa of broiler chickens are shown in Table 9. On the 21st day, compared with the control group, the mRNA expression level of interferon-gamma (*IFN-γ*) in the LPS group significantly increased (*p* < 0.05), and the expression level of interleukin-17 (*IL-17*) slightly increased, but the difference in changes was not significant (*p* > 0.05). Compared with the LPS group, the expression level of *IL-17* in the low-dose ALP group was significantly reduced (*p* < 0.05), while those of *IFN-γ* and *IL-17* in the high-dose ALP group showed a decreasing trend (*p* > 0.05). On the 42nd day, compared with the control group, the expression level of *IL-17* in the LPS group significantly increased (*p* < 0.05), and that of *IFN-γ* showed an increasing trend (*p* > 0.05). Compared with the LPS group, the expression levels of *IL-17* were significantly reduced in both the low- and high-dose ALP groups (*p* < 0.05), while that of *IFN-γ* showed a decreasing trend, but the difference in changes was not significant (*p* > 0.05).

### 3.8. Effects of Dietary Alkaline Phosphatase on the Gene Expression Levels of Tight Junction Proteins in the Jejunal Mucosa of Broilers Under Lipopolysaccharide Stress

The effects of different treatments on the gene expression level of tight junction protein in the jejunal mucosa of broiler chickens are shown in Table 10. On the 21st day, compared with the control group, there was no significant change in the expression levels of tight junction protein genes in the LPS group, with a slight decrease in the expression level of *Claudin-3*, but the difference in changes was not significant (*p* > 0.05). Compared with the LPS group, the expression levels of *Claudin-3* were significantly increased in the low- and high-dose ALP groups (*p* < 0.05). On the 42nd day, compared with the control group, the expression levels of *Occludin*, zonula occludens-1 (*ZO-1*), and zonula occludens-2 (*ZO-2*) in the LPS group were significantly reduced (*p* < 0.05). Compared with the LPS group, the expression level of *ZO-1* in the low-dose ALP group significantly increased (*p* < 0.05), and that of *ZO-2* showed an upward trend (*p* > 0.05). The expression levels of *ZO-1* and *ZO-2* in the high-dose ALP group were significantly increased (*p* < 0.05).

## 4. Discussion

Production performance is an important indicator for measuring the growth and development status and feed conversion efficiency of broiler chickens, and it is also a key basis for evaluating metabolic changes and nutritional regulation effects in broiler chickens under stress [1,2,3]. In this study, we found that LPS induction significantly reduced the average daily feed intake and average daily weight gain of broiler chickens, especially in the early stages after stress induction (1–21 days old). This is consistent with previous research findings that LPS-induced immune stress can inhibit appetite and reduce growth performance by activating pro-inflammatory responses and neuroendocrine pathways [3,6,7,25]. LPS, as an important pathogen-associated molecular pattern of Gram-negative bacteria, can activate the hypothalamic–pituitary–adrenal axis, leading to elevated levels of corticosterone and subsequently inhibiting feeding behavior and nutrient utilization efficiency [4,5,25]. In this study, the average daily gain of broiler chickens treated with LPS decreased by 11.59% compared to the control group throughout the experimental period (1–42 days), further confirming the inhibitory effect of LPS induction on growth performance. At the same time, in this study, we also found that adding ALP (especially at a dose of 1000 U/kg) to the diet significantly alleviated LPS-induced growth inhibition, restoring the production performance of broiler chickens to levels close to the control group. This result is consistent with the observed improvement effect of ALP on weight loss caused by inflammatory bowel disease in rodent models [26]. The mechanism may lie in ALP neutralizing LPS through dephosphorylation, blocking the TLR4-mediated inflammatory signaling pathway, thereby alleviating LPS inhibition of the growth axis [19]. At the same time, the decrease in inflammation levels reduces the body’s metabolic consumption, allowing more nutrients to be used for growth, thereby promoting the recovery of production performance [27].

Immunoglobulin is an important effector molecule of the adaptive immune response in the body, and changes in its level can reflect the humoral immune status of the body [28]. In this study, we found that LPS induction induced numerical decreases in serum IgA and IgG levels and a numerical increase in IgM levels in 21-day-old broilers, although none of these changes reached statistical significance. Therefore, no firm conclusion regarding humoral immune dysregulation can be drawn from these data. Following ALP supplementation, the high-dose ALP group showed a numerical increase in serum IgA and a numerical decrease in IgM, suggesting a potential modulatory effect of ALP on immunoglobulin profiles. However, these changes were also not statistically significant. Possible reasons for the lack of significance include the LPS dose (stress intensity), the choice of sampling time points, and individual variation among broilers; further studies are needed to clarify these factors [29].

Oxidative stress is one of the important mechanisms of LPS-induced intestinal injury [30]. MDA is the final product of lipid peroxidation [31]. In this study, we found that LPS stress significantly increased the content of MDA in the jejunal mucosa on the 21st day, indicating a possible exacerbation of lipid peroxidation and suggesting that LPS may have induced oxidative stress. Adding ALP to the diet showed a trend of reducing MDA content. More importantly, on the 42nd day of the experiment, ALP supplementation significantly increased the activity of CAT and GSH-Px in the jejunal mucosa. CAT and GSH-Px are important endogenous antioxidant enzymes in the body that can clear excess reactive oxygen species [32]. This suggests that ALP may alleviate LPS-induced oxidation by enhancing the endogenous antioxidant enzyme system [31,32,33]. At present, there is limited research on the role of ALP in antioxidant regulation in poultry. However, previous studies have shown that intestinal ALP can indirectly improve the body’s redox status by regulating gut microbiota, reducing endotoxin translocation, and alleviating intestinal inflammation [19,20,21,22]. Therefore, the antioxidant effect of ALP in this study may be closely related to its neutralization of LPS and improvement in intestinal barrier function.

Intestinal morphology is an important indicator for evaluating intestinal health [34]. In this study, we found that LPS significantly increased the crypt depth of the duodenum, jejunum, and ileum in broiler chickens, and reduced the villus-to-crypt ratio, indicating atrophic damage to the intestinal mucosa. The addition of ALP, especially at 21 days of age, significantly improved the structure of the ileum, and partial recovery trends were also observed in the duodenum and jejunum. This is consistent with the protective effect of ALP on intestinal morphology observed in weaned piglets and colitis mouse models [17,26]. ALP may maintain the integrity of intestinal structure by alleviating LPS-induced local inflammatory responses, regulating gut microbiota, and other pathways [19,20,21,22].

Inflammatory factors play a crucial role in LPS-induced immune stress [4,5,35]. LPS treatment significantly upregulated the mRNA expression of *IFN-γ* and *IL-17* in the jejunal mucosa of broiler chickens, while ALP addition significantly reduced *IL-17* expression and to some extent inhibited increases in *IFN-γ*. The inhibitory effect of ALP on the expression of inflammatory cytokine genes is closely related to its function of LPS dephosphorylation [17,18,19]. There are research reports that ALP can downregulate the expression of pro-inflammatory cytokines in mammalian models by inhibiting the TLR4/NF-κB signaling pathway. The results of this study are consistent with those reports [36,37].

The maintenance of intestinal barrier function depends on the normal expression of tight junction proteins [38]. In this study, we found that LPS treatment significantly downregulated the expression of *Occludin*, *ZO-1*, and *ZO-2* in the ileal mucosa of broiler chickens at 42 days of age, while ALP addition significantly upregulated the expression levels of these genes. This suggests that ALP may repair LPS-induced intestinal barrier damage by enhancing the expression of tight junction proteins. Previous studies have confirmed that intestinal ALP can stabilize tight junction structures in the intestine through dephosphorylation and inhibit LPS-induced barrier dysfunction. The results of this study are consistent with this [21,39]. After repairing the intestinal barrier function of broiler chickens, ALP also reduced the entry of LPS and other harmful substances into the bloodstream through the intestinal barrier, further alleviating systemic inflammatory response and oxidative stress levels, thereby alleviating the negative impact of LPS on growth performance [40].

From the perspective of dose–response, low-dose ALP (1000 U/kg) showed more stable and significant improvement effects on most indicators. There may be two reasons for this. On one hand, ALP neutralizes LPS through dephosphorylation. Under relatively fixed LPS stress intensity, a certain amount of ALP is sufficient in effectively clearing LPS from the intestine. However, excessive addition may interfere with the normal phosphorylation/dephosphorylation homeostasis in the intestine due to limited substrates and may not provide additional protective effects [17,18,41]. On the other hand, high-dose exogenous ALP may trigger adaptive regulation of the intestinal environment, triggering feedback regulatory mechanisms of the gut microbiota or host intestinal epithelial cells, thereby weakening its beneficial effects [42,43]. ALP exhibits a non-linear dose–response in alleviating LPS-induced immune stress, with stable and effective protective effects at low doses (1000 U/kg), providing important references for its rational application in production.

In this study, the expression of pro-inflammatory cytokines (such as TNF-α, IL-6, and IL-1β) or the activation status of the NF-κB and MAPK pathways in the jejunal mucosa was not examined. Future research is warranted to investigate these molecular events, thereby providing a better understanding of the mechanisms underlying the observed changes in intestinal health and growth performance.

## 5. Conclusions

In summary, adding dietary ALP to feed can effectively alleviate the negative effects of lipopolysaccharide stress on broiler production performance by improving intestinal morphology, inhibiting inflammatory response, and regulating the expression of tight junction protein genes in the intestine. The effect is more effective at low doses (1000 U/kg). Therefore, ALP can be used as a potential functional feed additive to alleviate immune stress in broiler chickens during production.

## Figures and Tables

**Table 1:** Ingredient composition and calculated nutrient levels of the basal diet (dry matter basis).

Feed Ingredients	1–21 Days (%)	22–42 Days (%)
Corn (CP 8.0%)	50.34	57.28
Broken rice (CP 10.4%)	5.76	4.00
Soybean meal (CP 44%)	36.85	29.85
Rapeseed meal (CP 35.7%)	0.00	2.00
Soybean oil	3.00	3.00
Calcium hydrogen phosphate (anhydrous)	1.10	0.95
Stone powder	1.10	1.15
Lysine (70%)	0.28	0.25
Methionine (50%)	0.28	0.25
Threonine (98%)	0.08	0.06
Salt	0.30	0.30
Choline chloride	0.15	0.15
Antioxidant	0.02	0.02
Vitamin premix ^1^	0.62	0.62
Micronutrient premix ^2^	0.12	0.12
Total	100.00	100.00
Estimated value of nutrient content ^3^		
Metabolizable energy (MJ/kg)	12.52	12.48
Crude protein (%)	20.84	18.76
Calcium (%)	0.88	0.80
Available phosphorus (%)	0.42	0.41
Lysine (%)	1.37	1.22
Methionine (%)	0.48	0.44
Methionine + cysteine (%)	0.82	0.77
Threonine (%)	0.89	0.81

^1^ The vitamin premix provided the following per kg of diets: vitamin A, 9000 IU; vitamin D_3_, 2400 IU; vitamin E, 45 IU; vitamin K_3_, 2 mg; thiamine, 1.6 mg; riboflavin, 6.5 mg; niacin, 50 mg; calcium pantothenate, 12 mg; pyridoxin, 3.25 mg; biotin, 0.14 mg; folic acid, 0.8 mg; and cobalamin, 0.015 mg. ^2^ The micronutrient premix provided the following per kg of diets: Fe, 67.5 mg; Cu, 12 mg; Mn, 95 mg; Zn, 90 mg; I, 1.35 mg; Se, 0.29 mg. ^3^ Calculated value.

**Table 2:** Primer sequence for RT-qPCR.

Gene Names ^1^	Accession Number	Primer (5′-3′) ^2^	Product Size (bp)
*IFN-γ*	NM_205149.2	F: AGCCGCACATCAAACACATA	150
		R: AAGTCGTTCATCGGGAGCTT	
*IL-17*	NM_204460.2	F: GAGAGCCTCTTCAAGAAAGC	199
		R: GAGTTCACGCACCTGGAATG	
*Claudin 2*	NM_001277622.1	F: ATGGTCTCTATGGGACTCCAGC	190
		R: CACCAGCTGCTGGGTGATG	
*Claudin 3*	NM_204202.2	F: ATGTCTATGGGGCTGGAGATC	109
		R: CAGCCAGCCCAGCACCGAC	
*Occludin*	NM_205128.1	F: ATGCCCCCCCCACGGGGTA	147
		R: CGCACGATGCAGATGGCG	
*ZO-1*	XM_040706827.2	F: GGACAGCCCAGATGGCCATAG	136
		R: TCAGACTTACACTACAGGTAGC	
*ZO-2*	NM_001396726.1	F: TTCATCATCAGAAGCCACTTTGAG	136
		R: CAGCCAACTTTCCAAGTTTGCC	
*β-actin*	NM_205518.2	F: GAGAAATTGTGCGTGACATCAAG	126
		R: CCTGAACCTCTCATTGCCAAT	

^1^ *IFN-γ*: interferon gamma; *IL-17*: Interleukin-17; *ZO-1*: Zonula occludens-1; *ZO-2*: Zonula occludens-2. ^2^ F: Forward; R: Reverse.

**Table 3:** Effects of different treatments on growth performance of broilers ^1^.

Items ^2^	Control	LPS	LPS + 1000 U/kg ALP	LPS + 5000 U/kg ALP	*p* Value
1–21 days					
IW, g	40.66 ± 0.33	40.84 ± 0.44	40.74 ± 0.34	40.81 ± 0.42	0.871
FW, g	656.77 ± 20.68 ^a^	594.12 ± 9.09 ^c^	632.38 ± 37.83 ^b^	612.25 ± 23.06 ^bc^	0.007
ADFI, g/d	41.81 ± 2.24 ^a^	38.58 ± 0.74 ^b^	39.98 ± 1.71 ^ab^	39.45 ± 1.56 ^b^	0.043
ADG, g/d	29.34 ± 0.97 ^a^	26.35 ± 0.45 ^c^	28.17 ± 1.79 ^ab^	27.21 ± 1.09 ^bc^	0.006
FCR	1.38 ± 0.02	1.40 ± 0.03	1.37 ± 0.03	1.40 ± 0.03	0.209
22–42 days					
FW, g	2041.45 ± 50.54 ^a^	1809.74 ± 124.56 ^b^	1983.40 ± 56.17 ^a^	1970.38 ± 31.67 ^a^	0.001
ADFI, g/d	122.98 ± 2.22 ^a^	110.19 ± 8.68 ^b^	120.88 ± 0.95 ^a^	119.00 ± 1.48 ^a^	0.002
ADG, g/d	65.94 ± 2.10 ^a^	57.89 ± 5.86 ^b^	64.33 ± 1.96 ^a^	64.67 ± 1.76 ^a^	0.007
FCR	1.88 ± 0.06	1.92 ± 0.03	1.89 ± 0.05	1.87 ± 0.07	0.617
1–42 days					
ADFI, g/d	82.39 ± 1.90 ^a^	74.39 ± 4.65 ^b^	80.43 ± 0.85 ^a^	79.23 ± 1.32 ^a^	0.001
ADG, g/d	47.64 ± 1.20 ^a^	42.12 ± 2.97 ^b^	46.25 ± 1.33 ^a^	45.94 ± 0.76 ^a^	0.001
FCR	1.71 ± 0.03	1.75 ± 0.01	1.72 ± 0.04	1.72 ± 0.04	0.514

^1^ *n* = 5; ^a b c^, different superscripts within the same row indicate significant differences (*p* < 0.05); ^2^ LPS: lipopolysaccharide; ALP: alkaline phosphatase; IW: initial weight; FW: final weight; ADFI: average daily feed intake; ADG: average daily gain; FCR: feed conversion ratio.

**Table 4:** Effects of different treatments on serum IgA, IgM, and IgG levels in broilers ^1^.

Items ^2^	Control	LPS	LPS + 1000 U/kg ALP	LPS + 5000 U/kg ALP	*p* Value
21 day					
IgA, pg/mL	2116.51 ± 420.10	1752.64 ± 200.17	1742.34 ± 384.13	1844.27 ± 348.94	0.324
IgM, μg/mL	26.31 ± 11.73	42.16 ± 17.08	38.60 ± 10.13	40.15 ± 30.05	0.559
IgG, ng/mL	75.59 ± 28.92	47.32 ± 12.86	59.57 ± 9.38	71.37 ± 34.96	0.279
42 day					
IgA, pg/mL	1103.10 ± 145.78	1115.46 ± 235.93	1208.10 ± 169.36	1195.35 ± 289.36	0.861
IgM, μg/mL	49.88 ± 21.88	44.41 ± 16.46	38.06 ± 4.64	48.41 ± 16.41	0.792
IgG, ng/mL	53.33 ± 15.05	44.37 ± 11.38	48.10 ± 1.62	47.12 ± 9.85	0.662

^1^ *n* = 5; ^2^ LPS: lipopolysaccharide; ALP: alkaline phosphatase; IgA: immunoglobulin A; IgM: immunoglobulin M; IgG: immunoglobulin G.

**Table 5:** Effects of different treatments on antioxidant enzyme activities in the jejunal mucosa of broilers ^1^.

Items ^2^	Control	LPS	LPS + 1000 U/kg ALP	LPS + 5000 U/kg ALP	*p* Value
21 day					
T-AOC, μmol/mg prot	0.13 ± 0.01	0.11 ± 0.02	0.14 ± 0.03	0.14 ± 0.03	0.222
SOD, U/mg prot	2.76 ± 0.38	2.55 ± 0.38	3.22 ± 0.28	2.90 ± 0.99	0.349
GSH-Px, U/mg prot	17.32 ± 3.85 ^a^	13.00 ± 1.88 ^ab^	11.59 ± 2.10 ^b^	15.77 ± 4.17 ^ab^	0.045
CAT, U/mg prot	22.68 ± 6.11	21.13 ± 5.44	24.01 ± 1.31	19.83 ± 8.41	0.692
MDA, nmol/mg prot	0.46 ± 0.18 ^b^	1.09 ± 0.39 ^a^	0.78 ± 0.11 ^ab^	0.91 ± 0.24 ^a^	0.014
42 day					
T-AOC, μmol/mg prot	0.17 ± 0.02	0.17 ± 0.05	0.17 ± 0.03	0.22 ± 0.02	0.066
SOD, U/mg prot	2.72 ± 0.31	3.01 ± 0.50	3.21 ± 0.28	2.90 ± 0.32	0.234
GSH-Px, U/mg prot	14.38 ± 3.25 ^b^	11.95 ± 3.29 ^b^	14.04 ± 3.52 ^b^	23.23 ± 5.89 ^a^	0.003
CAT, U/mg prot	24.01 ± 6.91 ^b^	26.02 ± 6.42 ^b^	42.31 ± 4.67 ^a^	25.41 ± 16.29 ^b^	0.028
MDA, nmol/mg prot	0.99 ± 0.20	1.18 ± 0.42	1.14 ± 0.39	1.06 ± 0.64	0.900

^1^ *n* = 5; ^a b^, different superscripts within the same row indicate significant differences (*p* < 0.05); ^2^ LPS: lipopolysaccharide; ALP: alkaline phosphatase; T-AOC: total antioxidant capacity; SOD: superoxide dismutase; GSH-Px: glutathione peroxidase; CAT: catalase; MDA: malondialdehyde.

**Table 6:** Effects of different treatments on the duodenal villus morphology in broilers ^1^.

Items ^2^	Control	LPS	LPS + 1000 U/kg ALP	LPS + 5000 U/kg ALP	*p* Value
21 day					
Villus height, μm	1389.29 ± 198.24	1134.87 ± 125.02	1278.23 ± 125.94	1232.65 ± 131.95	0.094
Crypt depth, μm	202.13 ± 26.43 ^b^	259.20 ± 24.12 ^a^	184.56 ± 33.51 ^b^	210.88 ± 8.96 ^b^	0.002
V/C ratio	6.96 ± 1.31 ^a^	4.41 ± 0.60 ^b^	7.11 ± 1.48 ^a^	5.85 ± 0.74 ^ab^	0.004
42 day					
Villus height, μm	1637.58 ± 271.70	1555.60 ± 194.44	1632.10 ± 160.85	1663.06 ± 259.91	0.893
Crypt depth, μm	245.17 ± 50.91	272.61 ± 35.50	277.54 ± 57.39	262.68 ± 28.92	0.693
V/C ratio	6.80 ± 1.06	5.75 ± 0.76	6.02 ± 0.88	6.40 ± 1.22	0.387

^1^ *n* = 5; ^a b^, different superscripts within the same row indicate significant differences (*p* < 0.05); ^2^ LPS: lipopolysaccharide; ALP: alkaline phosphatase; V/C ratio: villus-height-to-crypt-depth ratio.

**Table 7:** Effects of different treatments on the jejunum villus morphology in broilers ^1^.

Items ^2^	Control	LPS	LPS + 1000 U/kg ALP	LPS + 5000 U/kg ALP	*p* Value
21 day					
Villus height, μm	1189.19 ± 267.64	1072.17 ± 192.63	1178.30 ± 86.41	1179.61 ± 215.19	0.771
Crypt depth, μm	156.99 ± 29.96 ^b^	218.00 ± 10.82 ^a^	161.60 ± 15.70 ^b^	168.79 ± 38.71 ^b^	0.007
V/C ratio	7.57 ± 1.16 ^a^	4.90 ± 0.74 ^b^	7.36 ± 1.00 ^a^	7.07 ± 0.62 ^a^	0.001
42 day					
Villus height, μm	1287.30 ± 181.24	1193.13 ± 233.91	1202.16 ± 181.47	1399.37 ± 306.73	0.484
Crypt depth, μm	186.98 ± 22.31 ^b^	232.76 ± 21.00 ^a^	203.23 ± 18.52 ^ab^	223.39 ± 29.15 ^a^	0.028
V/C ratio	6.95 ± 1.16	5.15 ± 1.08	5.94 ± 0.91	6.24 ± 1.00	0.089

^1^ *n* = 5; ^a b^, different superscripts within the same row indicate significant differences (*p* < 0.05); ^2^ LPS: lipopolysaccharide; ALP: alkaline phosphatase; V/C ratio: villus-height-to-crypt-depth ratio.

**Table 8:** Effects of different treatments on the ileum villus morphology in broilers ^1^.

Items ^2^	Control	LPS	LPS + 1000 U/kg ALP	LPS + 5000 U/kg ALP	*p* Value
21 day					
Villus height, μm	1004.92 ± 63.89 ^b^	739.24 ± 117.69 ^c^	1168.84 ± 114.78 ^ab^	1189.29 ± 110.00 ^a^	<0.001
Crypt depth, μm	167.64 ± 21.02 ^b^	223.21 ± 24.94 ^a^	155.98 ± 7.33 ^b^	182.62 ± 14.31 ^b^	0.001
V/C ratio	6.00 ± 0.70 ^b^	3.31 ± 0.41 ^c^	7.49 ± 0.67 ^a^	6.51 ± 0.78 ^ab^	<0.001
42 day					
Villus height, μm	1161.57 ± 227.04	1081.35 ± 176.77	1183.99 ± 294.79	1210.28 ± 113.34	0.822
Crypt depth, μm	203.11 ± 40.73	245.82 ± 11.77	253.64 ± 65.46	231.35 ± 15.43	0.260
V/C ratio	5.81 ± 1.09 ^a^	4.38 ± 0.56 ^b^	4.69 ± 0.32 ^b^	5.24 ± 0.52 ^ab^	0.027

^1^ *n* = 5; ^a b c^, different superscripts within the same row indicate significant differences (*p* < 0.05); ^2^ LPS: lipopolysaccharide; ALP: alkaline phosphatase; V/C ratio: villus-height-to-crypt-depth ratio.

**Table 9:** Effects of different treatments on the mRNA expression levels of inflammatory cytokines in the jejunal mucosa of broilers ^1^.

Items ^2^	Control	LPS	LPS + 1000 U/kg ALP	LPS + 5000 U/kg ALP	*p* Value
21 day					
*IFN-γ*	1.06 ± 0.37 ^b^	1.60 ± 0.31 ^a^	1.69 ± 0.40 ^a^	1.37 ± 0.28 ^ab^	0.045
*IL-17*	1.14 ± 0.62 ^a^	1.29 ± 0.73 ^a^	0.35 ± 0.11 ^b^	0.63 ± 0.46 ^ab^	0.044
42 day					
*IFN-γ*	1.14 ± 0.62	1.98 ± 0.31	1.57 ± 0.48	1.39 ± 0.59	0.110
*IL-17*	1.08 ± 0.43 ^b^	3.58 ± 0.87 ^a^	1.09 ± 0.55 ^b^	1.36 ± 0.25 ^b^	<0.001

^1^ *n* = 5; ^a b^, different superscripts within the same row indicate significant differences (*p* < 0.05); ^2^ LPS: lipopolysaccharide; ALP: alkaline phosphatase; *IFN-γ*: interferon-gamma; *IL-17*: interleukin-17.

**Table 10:** Effects of different treatments on the mRNA expression levels of tight junction proteins in the jejunal mucosa of broilers ^1^.

Items ^2^	Control	LPS	LPS + 1000 U/kg ALP	LPS + 5000 U/kg ALP	*p* Value
21 day					
*Claudin-2*	1.09 ± 0.49	1.02 ± 0.36	1.12 ± 0.41	1.22 ± 0.31	0.878
*Claudin-3*	1.04 ± 0.32 ^b^	0.91 ± 0.27 ^b^	1.53 ± 0.26 ^a^	1.66 ± 0.29 ^a^	0.001
*Occludin*	1.01 ± 0.20	0.92 ± 0.13	0.95 ± 0.10	0.99 ± 0.15	0.770
*ZO-1*	1.02 ± 0.22	0.94 ± 0.23	1.07 ± 0.35	1.15 ± 0.25	0.674
*ZO-2*	1.03 ± 0.27	0.82 ± 0.25	1.16 ± 0.19	1.05 ± 0.25	0.208
42 day					
*Claudin-2*	1.04 ± 0.32	1.67 ± 0.90	1.49 ± 0.23	2.01 ± 1.00	0.214
*Claudin-3*	1.02 ± 0.23	0.91 ± 0.48	0.91 ± 0.44	1.04 ± 0.54	0.948
*Occludin*	1.01 ± 0.16 ^a^	0.48 ± 0.19 ^b^	0.48 ± 0.15 ^b^	0.59 ± 0.33 ^b^	0.004
*ZO-1*	1.00 ± 0.08 ^a^	0.61 ± 0.25 ^b^	0.95 ± 0.21 ^a^	0.93 ± 0.20 ^a^	0.022
*ZO-2*	1.06 ± 0.37 ^a^	0.55 ± 0.12 ^b^	0.67 ± 0.26 ^ab^	0.93 ± 0.33 ^a^	0.044

^1^ *n* = 5; ^a b^, different superscripts within the same row indicate significant differences (*p* < 0.05); ^2^ LPS: lipopolysaccharide; ALP: alkaline phosphatase; *ZO-1*: zonula occludens-1; *ZO-2*: zonula occludens-2.

## Data Availability

Raw data are retained by the author and may be available upon reasonable request.
